# Uniaxial, biaxial, and planar tension properties of deep fascia and a constitutive model to simultaneously reproduce these strain states

**DOI:** 10.3389/fbioe.2025.1494793

**Published:** 2025-04-07

**Authors:** Alejandro Aparici-Gil, Estefanía Peña, Marta M. Pérez

**Affiliations:** ^1^ Aragón Institute for Engineering Research (I3A), University of Zaragoza, Zaragoza, Spain; ^2^ Biomedical Research Networking Center in Bioengineering, Biomaterials and Nanomedicine (CIBER-BBN), Zaragoza, Spain; ^3^ Department of Anatomy, Embryology and Genetics, Veterinary Faculty, University of Zaragoza, Zaragoza, Spain

**Keywords:** fascia lata, constitutive models, material characterization, optimization, mechanical tests

## Abstract

This study aims to provide an in-depth analysis of the mechanical behavior of deep fascia through a comprehensive multidimensional characterization, including uniaxial, biaxial, and planar tension tests. To determine material parameters via test fitting, both a newly developed coupled exponential energy function and a previously proposed uncoupled exponential model—both considering two perpendicular fiber directions—are evaluated. For the uniaxial response, the mean stress measured was 3.96 MPa in the longitudinal direction and 0.6 MPa in the transverse direction at a stretch (λ) of 1.055. In planar tension tests, stress values of 0.43 MPa and 0.11 MPa were recorded for the longitudinal and transverse directions, respectively, at λ = 1.72. Under equibiaxial loading conditions, the mean stresses were 3.16 MPa and 1.2 MPa for the longitudinal and transverse directions when λ reached 1.037, respectively. The fitting results indicate that while the uncoupled exponential model effectively captures the uniaxial and equibiaxial experimental data, it fails to predict other mechanical responses accurately. In contrast, the coupled exponential strain energy function (SEF) demonstrates robust performance in both fitting and prediction. Additionally, an analysis was conducted to assess how the number and combination of tests influence the determination of material parameters. Findings suggest that a single biaxial test incorporating three loading ratios is sufficient to accurately capture and predict uniaxial, planar tension, and other biaxial strain states.

## 1 Introduction

The medical field is evolving due to computational technologies such as artificial intelligence, computational simulations, and extended reality. These technologies have the potential to guide processes and improve biomedical outcomes (Samant et al., 2023). Ramachandra et al. (2016) demonstrate how computational simulation can be used to study surgical procedures. It provides a powerful tool for simulating the hemodynamics and wall mechanics of grafts in patient-specific coronary artery bypass procedures. Additionally, it enables the characterization of variations in mechanical stimulus indices between arterial and venous surgeries (Ramachandra et al., 2016). Pavan et al. (2015) focus their study on fascia simulation using finite element analysis, which facilitates the interpretation of the correlation between alterations in the volume and pressure of muscle compartments and the deformation of the crural fascia.

Fascia is a tissue of great importance, yet it remains largely unexplored. It consists of collagenous connective tissue that surrounds and interpenetrates skeletal muscles, joints, organs, nerves, and vascular structures. Fascial tissue forms a whole-body, three-dimensional viscoelastic matrix that provides structural support (Klingler et al., 2014). According to Langevin and Huijing (2009), it is composed of three main structures: the superficial fascia, located directly beneath the skin, consisting of dense and areolar connective tissue along with fat; the deep fascia, a continuous sheet primarily made of dense, irregularly arranged connective tissue that restricts changes in the shape of underlying tissues; and muscle-related layers, characterized by irregularly arranged collagen fiber sheets that envelop muscles and may include both dense and areolar connective tissue layers.

Fascia forms a continuous network throughout the body and plays a crucial role in transmitting mechanical forces between muscles (Findley et al., 2012). Under basal tension from muscle insertions, the fascia maintains an inherent state of tension. When muscles contract, their insertions transmit a portion of the traction to the fascia, activating nerve endings embedded within its structure (Stecco et al., 2007), which provide essential sensory feedback to the brain about the body’s state. However, fascia is not merely a passive force transmitter. Schleip et al. (2019) found that fascial tissue exhibits a contractile response to different pharmacological agents, suggesting active behavior. Another key function of fascia is elastic energy storage, where energy accumulated during the stance phase is later released to propel the limb forward during the swing phase (Eng et al., 2014). Additionally, fascia helps regulate mechanical stress by absorbing, storing, and releasing kinetic energy (Zullo et al., 2017).

Concerning the mechanical behavior and biomechanics of fascia, it is known that fascia is an incompressible tissue; thus, the application of large displacement theory for incompressible, non-linear, and anisotropic materials should be employed (Findley et al., 2012). Its anisotropic behavior is attributed to the spatial orientation of collagen fibers, which vary along the sheet to ensure an appropriate response to mechanical demands. Like other soft tissues, fascia also exhibits viscoelastic properties, partly due to fluid movement within its solid matrix and the friction between its fluid and solid components (Peña et al., 2008).

To better understand fascia behavior under both normal and pathological conditions, as well as the relationship between structure and function, a numerical formulation capable of describing its mechanical properties is highly useful (Stecco et al., 2009). Several studies have been conducted to characterize these mechanical properties, including constitutive models that associate material properties with microstructure and parameters. Because different strain states exist, various testing protocols have been developed, such as uniaxial, biaxial, pure shear, and planar tension tests. Pavan et al. (2015) performed uniaxial tests and proposed a constitutive model for the crural fascia. Eng et al. (2014) and Pancheri et al. (2014) carried out biaxial and planar tests, respectively, proposing constitutive models based on the microstructure. However, these studies only considered a single strain state. Ruiz-Alejos et al. (2016) examined both uniaxial and pure shear properties, proposing a constitutive model that incorporates two strain states. However, this study did not include biaxial testing, and according to Sednieva et al. (2020), biaxial testing provides a more accurate representation of fascia loading than uniaxial or pure shear testing.

The present work aims to investigate in depth the mechanical behavior of the deep fascia through a multidimensional characterization, incorporating uniaxial (UT), biaxial (BxT), and planar tension (PT) tests. Although constitutive models for connective tissues, such as tendons and ligaments, already exist, the unique anatomical and histological characteristics of the fascia require adaptations to these models (Stecco et al., 2009). To determine material parameters through test fitting, we analyze a previously proposed uncoupled exponential-type strain energy function (SEF) (Pancheri et al., 2014) and introduce a newly proposed coupled SEF that accounts for two perpendicular fiber directions, following Stecco et al. (2009). Uncoupled structural models are unable to provide accurate fits when considering perpendicular anisotropic directions; therefore, a new coupled SEF is proposed based on Costa et al. (2001) and modified using invariants (Laita et al., 2024). In addition, we conducted a test combination study to identify the optimal set of experiments that yield parameters capable of both fitting and predicting different deformation states. The fitting process provides a parameter set that ensures that computational simulations can be performed with confidence, regardless of the deformation state being simulated.

## 2 Materials and methods

We propose three mechanical tests (UT, BxT, and PT) to reproduce the strain states in which the fascia primarily functions. Both selected constitutive models are structural models, which means that the model parameters are associated with the structural components of the tissue. Therefore, a relationship must exist between the parameter values and the physiological function of the corresponding tissue component. The two different SEFs are analyzed using the mean curves obtained from experimental tests. Finally, an analysis is performed to determine the number of tests needed for proper fitting and prediction.

### 2.1 Multidimensional characterization

The uniaxial tensile test is the most widely used method for material characterization (Calvo et al., 2010; Martins et al., 2010; Stecco et al., 2013). It provides stiffness measurements through Young’s modulus, and if the sample undergoes loading and unloading cycles, it also offers insights into viscoelastic properties (Peña et al., 2010). Soft biological tissues such as the arteries, heart, and fascia contain fibers oriented in different directions, forming their internal structure. As a result, their mechanical response varies depending on the loading direction (Guo et al., 2023; Ren et al., 2022; Eng et al., 2014). Biaxial tensile tests are commonly used to evaluate the mechanical anisotropy of these tissues (Takada et al., 2023). However, uniaxial or biaxial tests do not always fully characterize deformation states. In certain cases, tissue behavior cannot be solely described as uniaxial or biaxial, making it necessary to include planar tension tests. For example, Acosta Santamaría et al. (2015) investigated the mechanical behavior of the linea alba in the context of laparotomy closure using planar tension tests. For these reasons, in this work, a multidimensional characterization was conducted using UT, PT, and BxT to replicate the strain states in which the fascia primarily functions.

#### 2.1.1 Sample preparation

The fascia tissues were obtained from male sheep aged 1 year and harvested by veterinarians at the University of Zaragoza. The animals were sacrificed in a slaughterhouse for another study, which does not affect the results or the purpose of this work. After euthanasia (pentobarbital sodium, 8 mL), the fascia lata, attached to the aponeurosis of the tensor fasciae latae muscle, was removed. Once the fascia sheets were dissected, they were frozen at −20°C until the testing day. Previous experience from various experimental tests in our laboratory indicates that cryopreservation helps maintain mechanical properties. Our findings are supported by Stemper et al. (2007), who demonstrated that specimens preserved for 3 months using standard freezing techniques retained their physiological, subfailure, and rupture mechanical properties. The fascia sheet is thawed on the same day it is tested. Once it reaches room temperature, muscle and connective tissue residues are removed using a blade, and samples are cut.

A specific punch was designed for each test: for UT, a dog-bone punch with a central region of interest measuring 25 mm × 5 mm (5:1 aspect ratio), with 25 mm between clamps, was used. For PT, a rectangular punch with a 5 mm × 35 mm region of interest (1:7 aspect ratio) and a distance of 5 mm between the clamps was used. Finally, for BxT, a cruciform punch was chosen, with a central region of interest measuring 15 mm × 15 mm.

**FIGURE 1:**
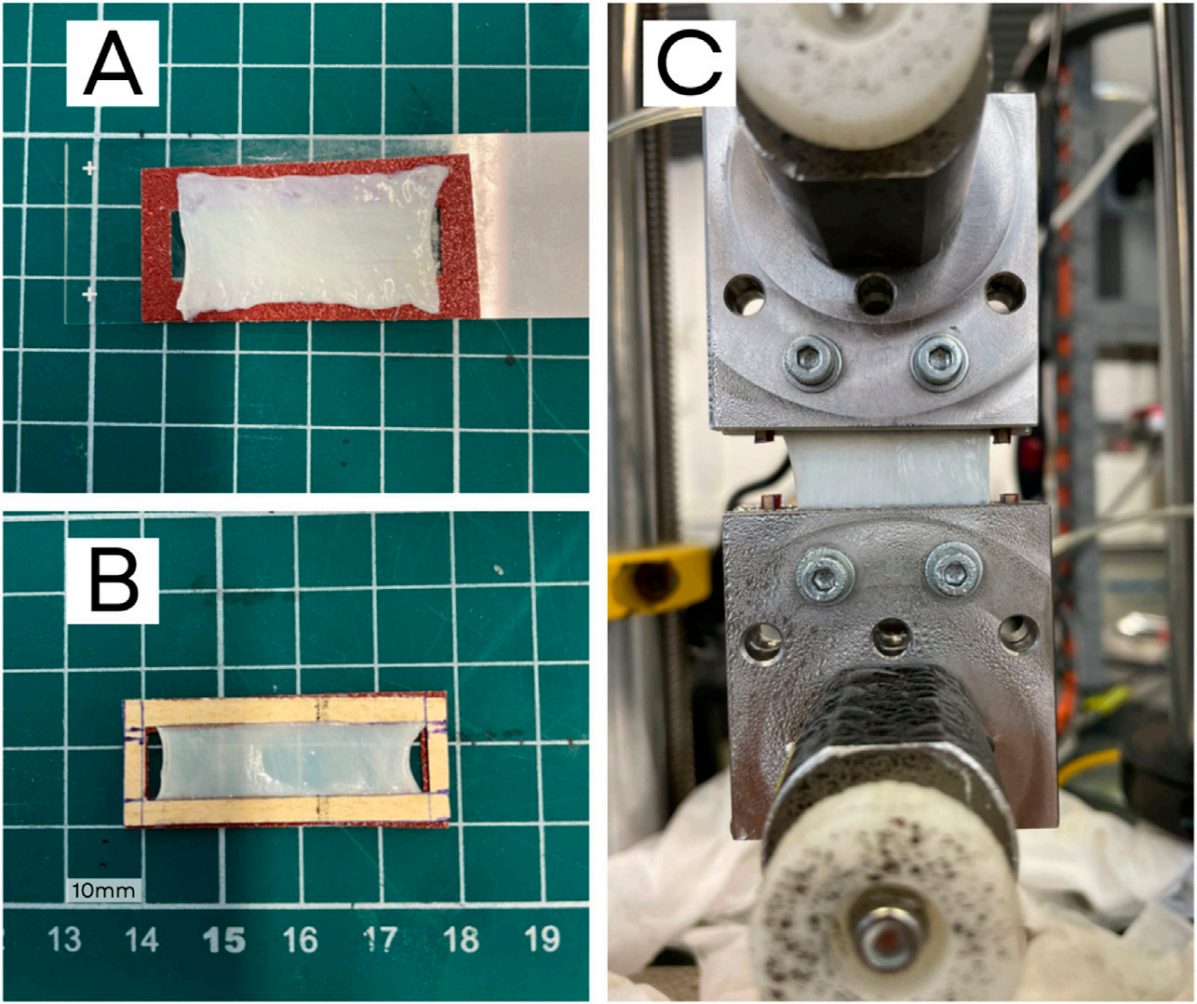
Preparation of a PT sample: **(A)** sample on a sandpaper frame, **(B)** sandpaper frame glued to the fascia, and **(C)** sample placed in the testing machine with pneumatic clamps and screws. The frame sides are cut prior to testing.

After cutting the samples, a black paint spray was applied to create randomized markers for tracking points and measuring the strain map. To prevent slippage between the fascia and clamps, sandpaper was fixed to the ends of the samples using cyanoacrylate glue (Loctite 401), as shown in Figure 1.

To avoid dehydration effects, UT and BxT tests were conducted while submerged in PBS solution (sodium chloride physiological solution, BioUltra tablet, Sigma-Aldrich GmbH). For PT, pneumatic clamps were required, so a humidifier was used to maintain proper hydration conditions, as the sample size prevented using a submerged testing chamber.

Following Stecco et al. (2009), the collagen fibers in adjacent fascia layers are oriented in two preferred directions, forming an angle between 80° and 90°. For our model, we assume a 90° orientation between anisotropy directions, referring to them as the longitudinal and transverse directions. When collecting samples, the longitudinal direction corresponds to the primary fiber alignment within the tissue. To ensure proper orientation, the punch’s longitudinal axis was aligned parallel to these macroscopically distinguishable fibers. We obtained samples in the transverse direction by rotating the punch 90° from this position.

#### 2.1.2 Histological analysis

**FIGURE 2:**
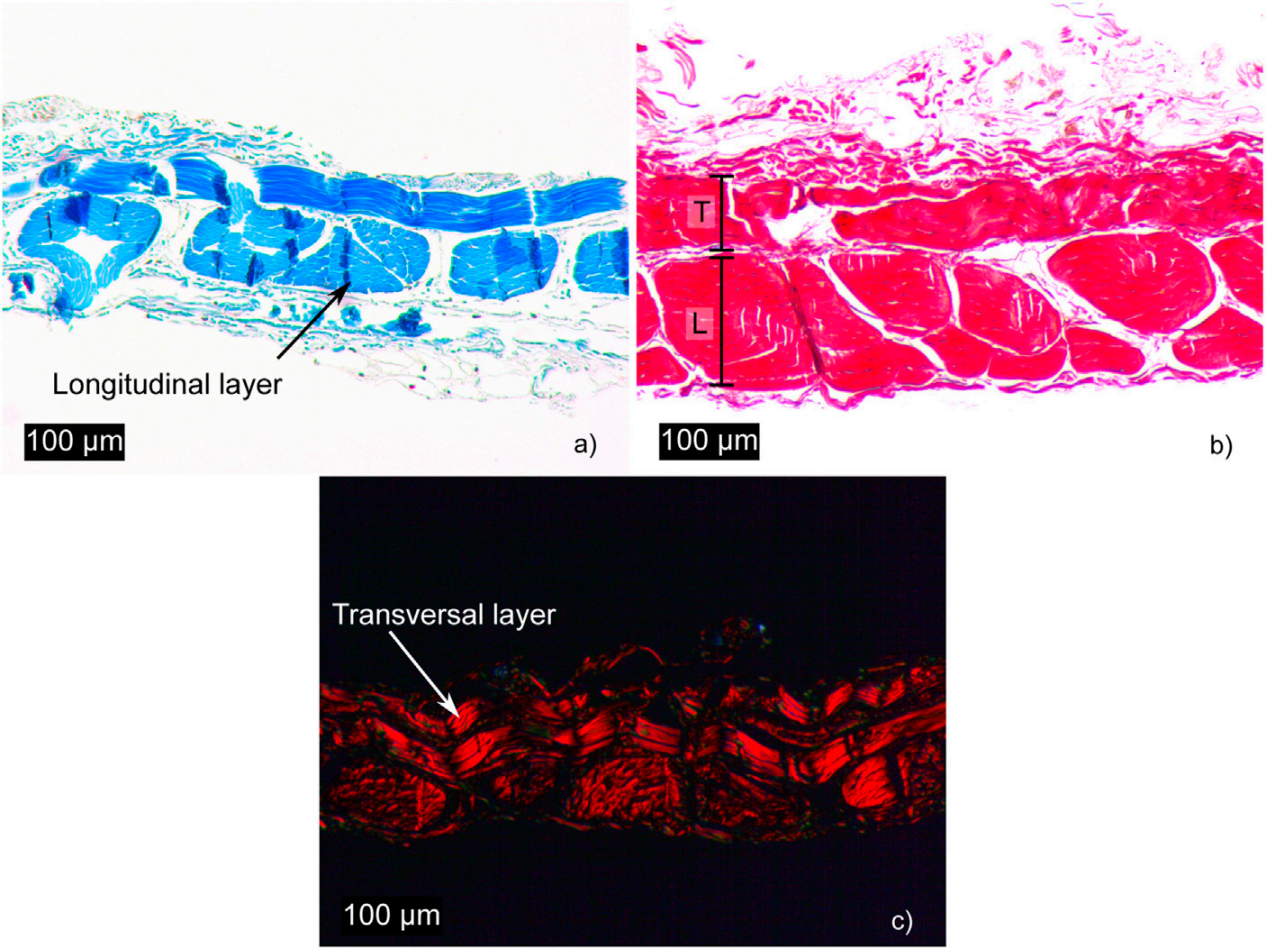
Histological sections of fascia: **(a, b)**, stained with Masson’s trichrome and Picrosirius Red, respectively, show the different collagen fiber densities in the longitudinal (L) and transverse (T) layers. **(c)**, stained with Picrosirius Red, reveals collagen fibers under polarized light.

Histological sections were analyzed using Masson’s trichrome (Figure 2A), where collagen appears in blue, and Picrosirius Red (Figures 2B, C), which, under polarized light, reveals collagen fibers in red-orange against a black background.

#### 2.1.3 Mechanical testing and protocols

Fourteen uniaxial tests were considered, seven for each direction, from a total of 15 longitudinal and 13 transverse samples to obtain the mean curve. In addition, 20 biaxial tests and 17 planar tension tests were performed—nine in the longitudinal direction and eight in the transverse direction, with six tests used to determine the mean curves for each strain state.

UT and PT followed the same protocol: three strain levels (2.5%, 5%, and 7.5%) with a strain rate of 10%/min were applied, subjecting the sample to five cycles at each level. After the last cycle was completed, the sample was stretched to rupture. The sample was first placed on the upper clamp, and a load balance was performed to compensate for the weight effect. The other end of the sample was then attached to the bottom clamp and stretched to achieve a 0 N load. Once at 0 N, the chamber was filled with PBS, and a second load balance was conducted to compensate for the fluid effect before stretching the sample to the pre-load level.

UT tests were performed using the Instron MicroTester 5548, equipped with steel clamps and a 50 N load cell with a sensitivity of ± 0.025% of the measured load. The pre-load level was set at 0.08 N, following Pancheri et al. (2014). For PT, the Instron MicroTester 5848 was used, featuring pneumatic steel clamps and a 50 N load cell. A pre-load value of 1.5 N was chosen to ensure a proper initial state.

For the biaxial protocol, a strain level of 10% and a strain rate of 20%/min were applied, along with five loading ratios: 1:1, 0.5:1, 1:0.5, and 0.75:1, denoted as E1, E2, E3, E4, and E5, respectively. The first value of each ratio corresponds to the longitudinal direction. Ratios E1 to E3 were used to fit the material parameters, while E4 and E5 were employed to evaluate the predictive capability of the constitutive model. Each ratio was tested over five cycles. Biaxial tests were conducted using the Instron Planar Biaxial Soft Tissue Test System, equipped with four 50 N load cells. Steel clamps were used, with sandpaper glued to the sample using Loctite 401 and secured with screws to prevent slippage between the sample and the clamps. According to Vitucci (2024), the sample geometry can lead to errors. However, this phenomenon was studied by Cilla et al. (2019), suggesting that our geometry and clamped system leads to shear stresses in the central region close to zero. A pre-load value of 0.5 N was established.

**FIGURE 3:**
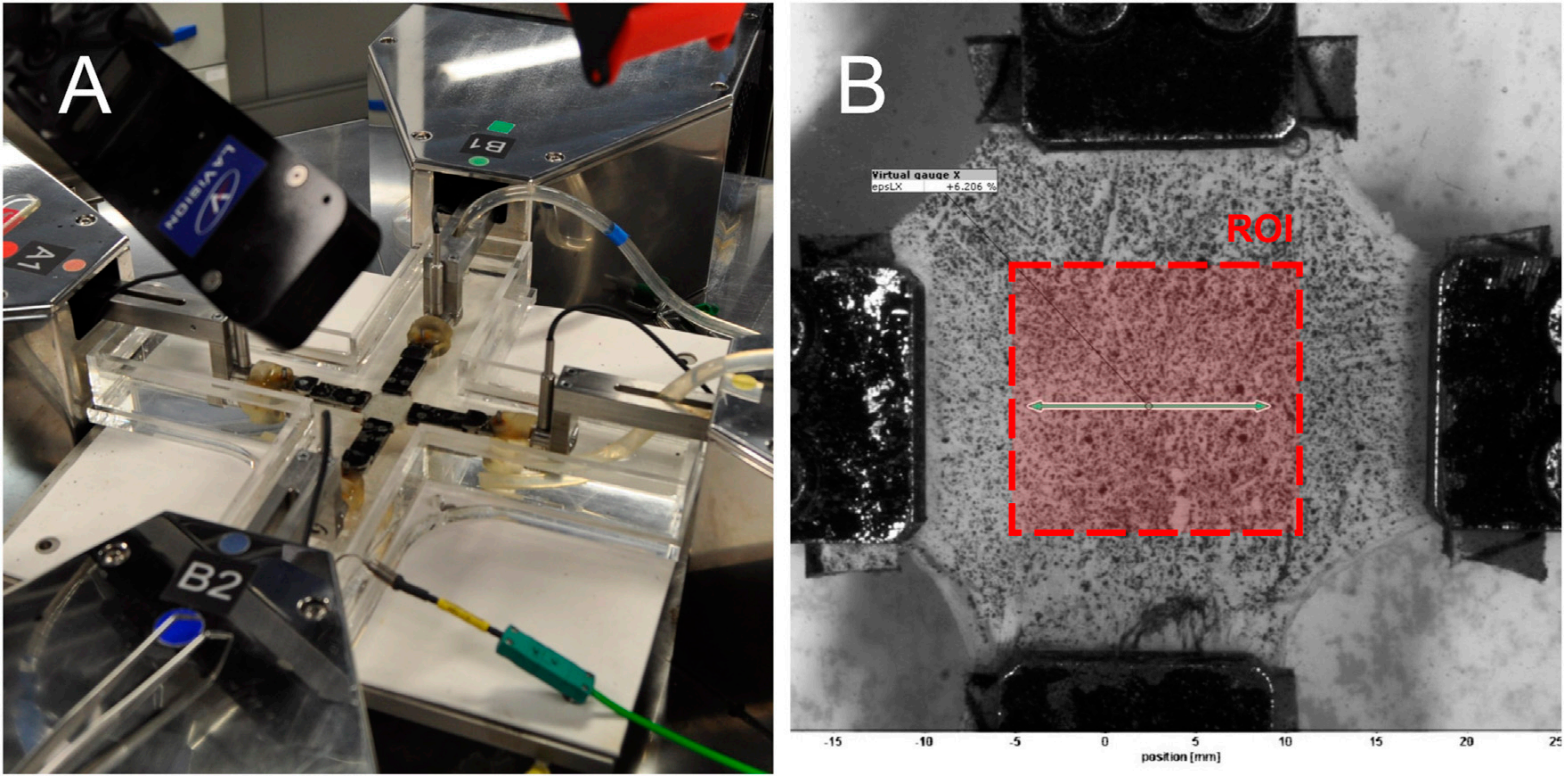
Biaxial testing setup: **(A)** Instron Planar Biaxial Soft Tissue Test System during a test and **(B)** image from DIC analysis. Note that the region of interest for strain calculation is defined by the area corresponding to the width of the clamps.

UT and BxT tests were recorded at a frame rate of 3 Hz using the LaVision camera system. The acquired images were processed using the free version of GOM Correlate, a digital image correlation (DIC) software for tracking patterns and computing displacements and deformations. A virtual gauge was defined, as shown in Figure 3B, and strain values were obtained from this gauge. The initial position and length of the virtual gauge were kept consistent across all tests to minimize potential sources of error and variability. In soft tissues, the displacement between clamps is typically larger than in the central region. Because the formulation is valid only in the central region, DIC was necessary to accurately measure deformations in the region of interest. For PT, the DIC system was not used because the distance between clamps was small, making it reasonable to assume that clamp displacement corresponded to the displacement of the region of interest.

### 2.2 Constitutive models

Soft tissues are usually modeled as composite materials consisting of an isotropic base material reinforced by collagen fibers aligned in two different directions (Peña et al., 2010).

To ensure an accurate reproduction of the fascia’s mechanical response, two material models have been considered (Laita et al., 2024). The first model, based on Holzapfel et al. (2000) and proposed by Pancheri et al. (2014), assumes exponential uncoupled volumetric-deviatoric responses and has been widely used to describe the mechanical behavior of fiber-reinforced soft tissues (Peña et al., 2010; Calvo et al., 2010; Eng et al., 2014). The second model, proposed herein, is a modified exponential invariant-based version of the Costa model (Costa et al., 2001), as introduced by Laita et al. (2024), which considers a coupled response. Within the framework of hyperelasticity, both models assume the tissue is incompressible, undergoes large displacements, and exhibits non-linear anisotropic behavior.

#### 2.2.1 Fundamental equations

An arbitrary point identified by its position vector, X , belonging to an undeformed configuration called reference configuration, $B_{r}$ , is chosen. The external mechanical forces deform $B_{r}$ , therefore, X has a new position $x=\chi \left(X\right)$ belonging to the deformed configuration B . The deformation of the body is described by the vector field χ , which assigns to points X a particular position x in B and attributes a particular reference position X in $B_{r}$ to each point x (Holzapfel et al., 2000).

Following the standard notation, we call F the deformation gradient tensor relative to $B_{r}$ and define it as $F=\nabla \chi \left(X\right)$ , with the Cartesian components $F_{iJ}=\partial x_{i}/\partial X_{J}$ with i,J∈{1,2,3} . J is the determinant of the deformation gradient tensor F representing the local volume ratio. The left and right Cauchy–Green deformation tensors are defined as $B=FF^{\text{T}}$ and $C=F^{\text{T}}F$ , respectively.

The theory of hyperelasticity describes the elastic behavior of a body through a strain energy function, denoted as Ψ , which is defined per unit volume in the reference configuration $B_{r}$ . This work assumes an incompressible material, hence J=det F≡1 . The first Piola–Kirchhoff tensor P and the Cauchy stress tensor σ are given by Equation 1:

$$P=\frac{\partial \Psi }{\partial F}-pF^{-1}\;\sigma =F\frac{\partial \Psi }{\partial F}-pI,$$ (1)

where p is the hydrostatic pressure. The two directions of anisotropy are given by the unit vectors M and N in the undeformed configuration $B_{r}$ . Structural tensors are defined, following Spencer (1971) and Ogden (2001), as M⊗M and N⊗N . Then, the form of Ψ is reduced to the dependence on the principal invariants $I_{1},I_{2},I_{3}$ of C and $I_{4},I_{5},I_{6},I_{7},I_{8}$ of M and N . Based on the structure of fascia and following the simplification suggested by Holzapfel et al. (2000), we reduce the number of invariants to $I_{1},I_{4},I_{6}$ . Therefore, the expression of the Cauchy stress tensor is reduced to Equation 2:

$$\sigma =2\Psi_{1}B+2\Psi_{4}m⊗m+2\Psi_{6}n⊗n-pI,$$ (2)

where $\Psi_{i}=\partial \Psi /\partial I_{i}$ with i∈{1,4,6} , m=FM , n=FN , and invariants are defined as follows: $I_{1}=trC$ , $I_{4}=M\cdot \left(CM\right)$ , and $I_{6}=N\cdot \left(CN\right)$.

Following experimental observations in Section 2.1.1, this work considers a 90° angle between anisotropy directions; thus, unit vectors are defined as

$$M=\left\{1,0,0\right\}\;N=\left\{0,1,0\right\}.$$

For planar tissue, components of the deformation gradient F can be expressed by Equation 3:

$$F=\left[\begin{array}{ccc} F_{11} & F_{12} & 0\\ F_{21} & F_{22} & 0\\ 0 & 0 & F_{33} \end{array}\right].$$ (3)

Finally, for each deformation state, and assuming incompressibility $\left(\lambda_{1}\lambda_{2}\lambda_{3}=1\right)$ , the deformation gradient tensor is given by Equation 4:

$$F_{\mathrm{UT}}=\left[\begin{array}{ccc} \lambda_{i} & 0 & 0\\ 0 & 1/\sqrt{\lambda_{i}} & 0\\ 0 & 0 & 1/\sqrt{\lambda_{i}} \end{array}\right]\;F_{\mathrm{PT}}=\left[\begin{array}{ccc} \lambda_{i} & 0 & 0\\ 0 & 1 & 0\\ 0 & 0 & 1/\lambda_{i} \end{array}\right]\;F_{\mathrm{BxT}}=\left[\begin{array}{ccc} \lambda_{1} & 0 & 0\\ 0 & \lambda_{2} & 0\\ 0 & 0 & 1/\lambda_{1}\lambda_{2} \end{array}\right],$$ (4)

where i=1,2 1 refers to the longitudinal direction, while 2 refers to the transversal direction.

#### 2.2.2 Uncoupled strain energy function

The uncoupled SEF based on Pancheri et al. (2014) is expressed as a combination of two parts: one related to the homogeneous properties of the substrate material and the other to the anisotropy resulting from the included fibers. It follows Equation 5:

$$\Psi =\Psi_{\mathrm{iso}}+\Psi_{\mathrm{aniso}}=\Psi_{\mathrm{iso}}\left(I_{1}\right)+\Psi_{fib_{4}}\left(I_{4}\right)+\Psi_{fib_{6}}\left(I_{6}\right).$$ (5)

The isotropic contribution of the matrix, $\Psi_{\mathrm{iso}}$ , is modeled following the Demiray exponential strain energy function (Demiray, 1972) expressed by Equation 6:

$$\Psi_{\mathrm{iso}}=\frac{\mu_{\mathrm{iso}}}{2\alpha }\left\{exp\left[\alpha \left(I_{1}-3\right)\right]-1\right\},$$ (6)

where $\mu_{\mathrm{iso}}$ is a positive stress-like parameter and α is a dimensionless material parameter.

The anisotropic part of the model, $\Psi_{\mathrm{aniso}}$ , also follows an exponential strain form; it has two uncoupled terms, one related to the longitudinal direction (l) and the other to the transverse direction (t) and is expressed by Equation 7:

$$\Psi_{\mathrm{fib}}=\frac{\mu_{l}}{2k_{l}}\left\{exp\left[k_{l}{\left(I_{4}-1\right)}^{2}\right]-1\right\}+\frac{\mu_{t}}{2k_{t}}\left\{exp\left[k_{t}{\left(I_{6}-1\right)}^{2}\right]-1\right\}.$$ (7)

The parameters $\mu_{\mathrm{iso}}$ , $\mu_{l}$ , and $\mu_{t}$ are positive stress-like parameters; α , $k_{l}$ , and $k_{t}$ are dimensionless parameters. $\mu_{l}$ and $\mu_{t}$ quantify the level of anisotropy, while $k_{l}$ and $k_{t}$ are associated with the respective directions.

According to Equation 2 and following the definition for $\Psi_{i}$ , we obtain Equation 8:

$$\Psi_{1}=\frac{\mu_{\mathrm{iso}}}{2}exp\left[\alpha \left(I_{1}-3\right)\right]\;\Psi_{4}=\mu_{l}exp\left[k_{l}{\left(I_{4}-1\right)}^{2}\right]\left(I_{4}-1\right)\;\Psi_{6}=\mu_{t}exp\left[k_{t}{\left(I_{6}-1\right)}^{2}\right]\left(I_{6}-1\right).$$ (8)

We denote this model as uncoupled because the derivatives of Ψ with respect to $I_{i}$ , $\Psi_{i}$ , depend only on $I_{i}$ , Equation 8.

#### 2.2.3 Coupled strain energy function

The proposed coupled SEF is based on the one proposed by Costa et al. (2001) and Laita et al. (2024) for myocardial tissue and is given by Equation 9:

$$\Psi =C_{0}\left(e^{Q}-1\right),$$ (9)

where $C_{0}$ is a positive stress-like parameter, and Q is the exponent of the exponential function that includes the isotropic and anisotropic character. This work proposes Q as the sum of three terms: a linear term for the isotropic matrix contribution and two quadratic terms related to the anisotropy directions. Thus, Q is defined as Equation 10:

$$Q=C_{1}\left(I_{1}-3\right)+C_{2}{\left(I_{4}-1\right)}^{2}+C_{3}{\left(I_{6}-1\right)}^{2},$$ (10)

with $C_{1},C_{2},C_{3}$ being dimensionless parameters. The quadratic term dependent on $I_{4}$ represents the longitudinal fiber direction, while the term dependent on $I_{6}$ is associated to the transverse direction. Following Equation 2 shown before, the terms $\Psi_{1}$ , $\Psi_{4}$ , and $\Psi_{6}$ are given by Equation 11:

$$\Psi_{1}=C_{0}C_{1}e^{Q}\Psi_{4}=C_{0}C_{2}e^{Q}\left(2I_{4}-2\right)\;\Psi_{6}=C_{0}C_{3}e^{Q}\left(2I_{6}-2\right).$$ (11)

We denote our proposed SEF as coupled due to the terms $\Psi_{i}$ depending on all invariants that are associated with the isotropic and anisotropic contributions through $e^{Q}$ .

### 2.3 Fitting procedure, combination of tests, and model comparison

A MATLAB script was developed to analyze the optimal combination of tests and optimize the fitting process. Five types of tests were available for fitting (UT, PT, E1, E2, and E3). The number of tests to combine could be chosen while leaving the rest for prediction, in addition to E4 and E5 biaxial ratios. In this way, combinations of three tests were conducted for both uncoupled and coupled models to study the structural parameters obtained by fitting. The model that provides the best fitting and prediction was chosen to study the combinations with different numbers of tests involved.

Given p , a vector of the q unknown parameters of the SEF, the referred minimization problem can be stated as Equation 12:

$$\underset{p}{\min}\|\chi \left(p\right){\|}_{2}^{2}=\underset{p}{\min}\left(\overset{N}{\underset{i=1}{\Sigma }}\left[{\left(\sigma_{i}-\sigma_{1}^{\Psi }\right)}^{2}\right]\right),$$ (12)

where N is the number of considered points, σ is the stress computed from the experimentally measured force, $\sigma^{\Psi }$ is the analytical stress, q is the number of parameters of the SEF, and the overlined symbols refer to the mean.

For choosing the proper combination $p^{*}$ , we analyze the R-square error, $R^{2}$ , the root mean square error (RMSE), ε , and the relative error $err^{*}$ (Destrade et al., 2017) of the fit and predictive processes for all possible combinations, as described in Equation 13:

$$R^{2}=1-\frac{\sum_{i=1}^{N}{\left(\sigma_{i}-\sigma_{i}^{\Psi }\right)}^{2}}{\sum_{i=1}^{N}{\left(\sigma_{i}^{\Psi }-\bar{\sigma^{\Psi }}\right)}^{2}}\;\varepsilon =\frac{\sqrt{\frac{\sum_{i=1}^{N}{\left(\sigma_{i}-\sigma_{i}^{\Psi }\right)}^{2}}{N-q}}}{\bar{\sigma }}\;err^{*}=\underset{i}{\max}\left|\left(\frac{\sigma_{i}-\sigma^{\Psi }\left(p^{*}\right)}{\sigma_{i}}\right)\right|.$$ (13)

Following the incompressibility hypothesis $\left(\lambda_{1}\lambda_{2}\lambda_{3}=1\right)$ , the analytical expressions for the non-null Cauchy stress terms obtained from our proposed coupled exponential SEF for the biaxial strain state are described by Equations 14, 15:

$$\sigma_{l}=-\frac{2\;C_{0}\;e^{C_{1}\left(I_{1}-3\right)+C_{2}{\left(I_{4}-1\right)}^{2}+C_{3}{\left(I_{6}-1\right)}^{2}}\left(C_{1}-C_{1}\;\lambda_{l}^{4}\;\lambda_{t}^{2}+2\;C_{2}\;\lambda_{l}^{4}\;\lambda_{t}^{2}-2\;C_{2}\;I_{4}\;\lambda_{l}^{4}\;\lambda_{t}^{2}\right)}{\lambda_{l}^{2}\;\lambda_{t}^{2}},$$ (14)

$$\sigma_{t}=-\frac{2\;C_{0}\;e^{C_{1}\left(I_{1}-3\right)+C_{2}{\left(I_{4}-1\right)}^{2}+C_{3}{\left(I_{6}-1\right)}^{2}}\left(C_{1}-C_{1}\;\lambda_{l}^{2}\;\lambda_{t}^{4}+2\;C_{3}\;\lambda_{l}^{2}\;\lambda_{t}^{4}-2\;C_{3}\;I_{6}\;\lambda_{l}^{2}\;\lambda_{t}^{4}\right)}{\lambda_{l}^{2}\;\lambda_{t}^{2}}.$$ (15)

In the case of a uniaxial strain state, the analytical expressions are given by Equations 16, 17:

$$\sigma_{l}=-\frac{2\;C_{0}\;e^{C_{1}\left(I_{1}-3\right)+C_{2}{\left(I_{4}-1\right)}^{2}+C_{3}{\left(I_{6}-1\right)}^{2}}\left(C_{1}-C_{1}\;\lambda_{l}^{3}+2\;C_{2}\;\lambda_{l}^{3}-2\;C_{2}\;I_{4}\;\lambda_{l}^{3}\right)}{\lambda_{l}},$$ (16)

$$\sigma_{t}=-\frac{2\;C_{0}\;e^{C_{1}\left(I_{1}-3\right)+C_{2}{\left(I_{4}-1\right)}^{2}+C_{3}{\left(I_{6}-1\right)}^{2}}\left(C_{1}-C_{1}\;\lambda_{t}^{3}+2\;C_{3}\;\lambda_{t}^{3}-2\;C_{3}\;I_{6}\;\lambda_{t}^{3}\right)}{\lambda_{t}}.$$ (17)

Finally, for the planar tension strain state, the expressions are given by Equations 18, 19:

$$\sigma_{l}=-\frac{2\;C_{0}\;e^{C_{1}\left(I_{1}-3\right)+C_{2}{\left(I_{4}-1\right)}^{2}+C_{3}{\left(I_{6}-1\right)}^{2}}\left(C_{1}-C_{1}\;\lambda_{l}^{4}+2\;C_{2}\;\lambda_{l}^{4}-2\;C_{2}\;I_{4}\;\lambda_{l}^{4}\right)}{\lambda_{l}^{2}},$$ (18)

$$\sigma_{t}=-\frac{2\;C_{0}\;e^{C_{1}\left(I_{1}-3\right)+C_{2}{\left(I_{4}-1\right)}^{2}+C_{3}{\left(I_{6}-1\right)}^{2}}\left(C_{1}-C_{1}\;\lambda_{t}^{4}+2\;C_{3}\;\lambda_{t}^{4}-2\;C_{3}\;I_{6}\;\lambda_{t}^{4}\right)}{\lambda_{t}^{2}}.$$ (19)

## 3 Results

### 3.1 Histological results

The longitudinal layer is characterized by a high density of collagen fibers forming fascicles, whereas the transverse layer is thinner, as illustrated in Figure 2A. The results demonstrate that fascia is a highly organized tissue with a clearly defined bilayered structure, as shown in Figure 2B. These layers intersect at an angle of approximately 90°. It can be observed that the transverse layer contains only a single row of collagen fibers, a finding consistent with Pancheri et al. (2014). Figure 2C, stained with Picrosirius Red and observed under polarized light, highlights the nearly 90-degree angle between the layers.

### 3.2 Mechanical experiments

**FIGURE 4:**
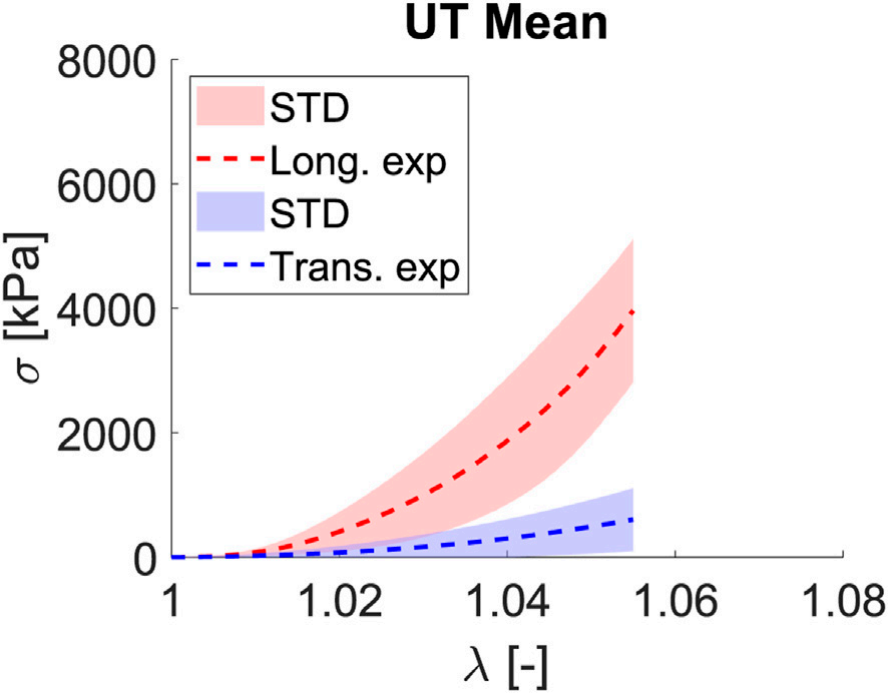
Mean and STD (shaded) for Cauchy stress σ [kPa] and stretch λ [−] curves for the longitudinal (red) and transverse (blue) fibers subjected to a uniaxial test.

Fascia lata, which surrounds the principal muscles of limbs, works preferentially along one direction, with most of the collagen fibers following this preferred direction, which we denoted as longitudinal; hence, the matrix and fiber transversal direction will play a secondary role in the mechanics and functionality of the fascia. Proof of this is the curves for the uniaxial tests shown in Figure 4. For a stretch of λ=1.055 , the longitudinal behavior is totally different from transverse, while $\sigma_{1}$ has an average stress value of 3.96 ± 1.15 MPa (mean ± STD), $\sigma_{2}$ only achieves a value of 0.60 ± 0.50 MPa. Following the mechanical behavior that soft tissues usually exhibit, the test begins with an initial zone with no stress increment, and then a strain increment appears (toe region). This is because the unfolding fibers are being stretched; when a value of λ=1.020 is reached, an exponential increase in stress values is experienced.

**FIGURE 5:**
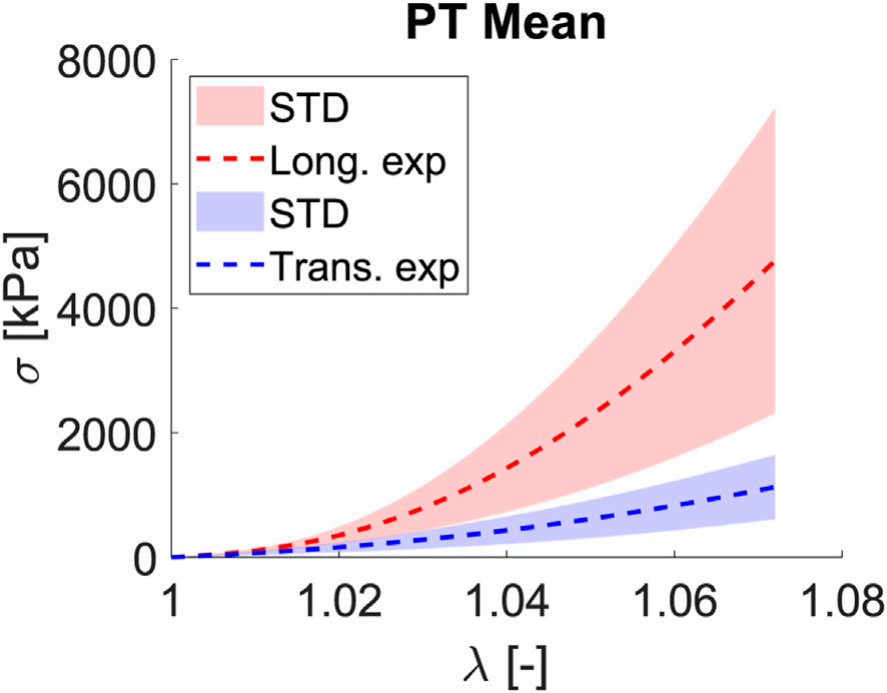
Mean and STD (shaded) for Cauchy stress σ [kPa] and stretch λ [−] curves for the longitudinal (red) and transverse (blue) fibers subjected to a planar tension test.

The planar tension test uses a large aspect ratio between width and length to measure shear properties. According to Moreira and Nunes (2013), for small deformations, the stress–stretch response for planar tension and simple shear is the same. Nevertheless, a divergence between planar tension and simple shear occurs for stretch values greater than 1.30. As we are far from λ values of 1.30, we consider planar tension valid for measuring simple shear properties. Curves for planar tension shown in Figure 5 describe a mechanical behavior with a longitudinal direction that exhibits greater stiffness in contrast to the transversal direction of fibers, as we observed in the uniaxial test. Longitudinal stress values are 4.77 ± 2.45 MPa (mean ± STD), whereas in the transversal direction, we observed 1.13 ± 0.51 MPa for a λ value of 1.072. A less pronounced non-linear behavior is observed compared to uniaxial curves. Regarding the deviation of the longitudinal curves from the mean, it has been noted that planar tension exhibits greater dispersion.

**FIGURE 6:**
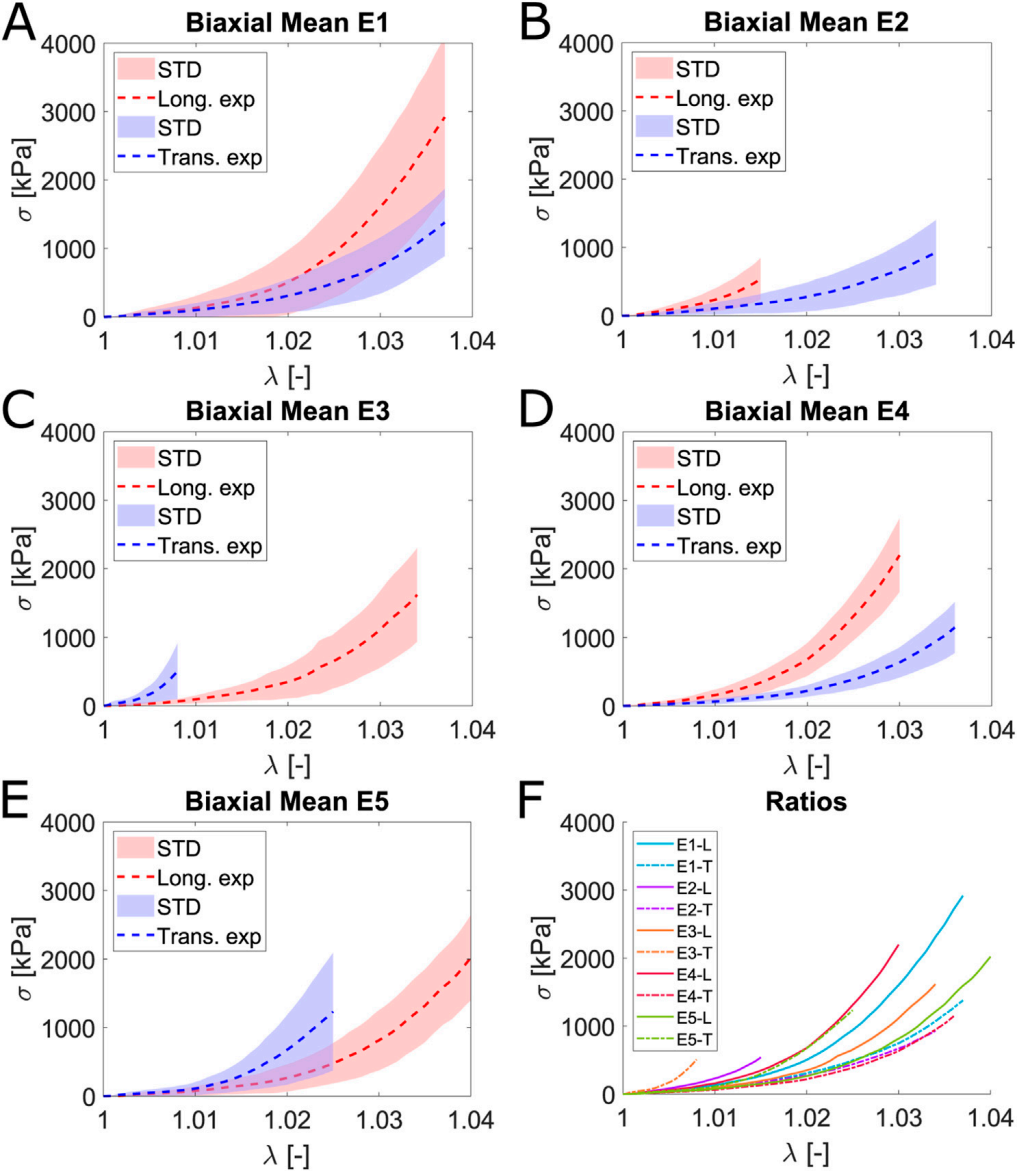
Mean and STD (shaded) Cauchy stress σ [kPa] and stretch λ [−] curves for the longitudinal (red) and transverse (blue) fibers subjected to different ratios in the biaxial test. **(A)** corresponds to the equibiaxial (1:1) ratio, while the curves in **(B)** show the ratio 0.5:1; **(C)**, **(D)**, and **(E)** correspond to the ratios 1:0.5, 0.75:1, and 1:0.75, respectively. **(F)** represents the mean Cauchy stress and stretch curves for both longitudinal and transverse fibers across all ratios.

The mean curves depicted in Figure 6 correspond to the last load cycle at each ratio for biaxial tests. The equibiaxial ratio (1:1) exhibits greater stiffness in both the longitudinal and transverse directions than in uniaxial and planar tension tests. Stretching one fiber family implies an increase in the stiffness of the other. Evidence of this effect is clearly observed by comparing the E1 and E4 ratios: Using the equibiaxial as a reference and considering the described effect of the ratios, a greater stretch in one direction leads to a stiffer curve in the opposite direction than the equivalent curve in the equibiaxial ratio, ensuring the proper performance of the biaxial test. This can be observed in Figure 6F, where the mean curves for each direction and ratio are presented.

Table 1 compiles the mean maximum stress and strain values for each direction and ratio obtained. We include the anisotropy ratio $\left(\eta =\sigma_{l}/\sigma_{t}\right)$ , defined as the ratio between the longitudinal stress value and the transverse stress value for a specific λ value. In order to compare η across the equibiaxial, uniaxial, and planar strain states, a stretch value of 1.037 has been chosen as a reference.

**TABLE 1:** Mean value for σ (mean ± STD) and η for equibiaxial, uniaxial, and planar tension strain states at a stretch value of 1.037.

Test	$\sigma_{1}$ [MPa]	$\sigma_{2}$ [MPa]	η [−]
Equibiaxial	2.92 ± 1.17	1.38 ± 0.49	2.12
Uniaxial	1.58 ± 0.92	0.26 ± 0.27	6.08
Planar tension	1.22 ± 0.59	0.38 ± 0.19	3.21

The η for the uniaxial test exhibits the highest value, of 6.08, followed by the η of the planar tension test, which reaches 3.21 and finally, the equibiaxial, where we found a η value of 2.12. The obtained values for η are reasonable given the characteristics of the different strain states, as the equibiaxial test involves both directions. As observed in Figure 6, increased stretching in one direction results in a stiffer curve in the opposite direction. Evidence of this is that the maximum transversal stress, $\sigma_{2}$ , for λ equal to 1.037 is obtained with the equibiaxial test.

A common point observed in all tests is the significant deviation found in the experiments. Two factors contributing to this could be the extraction area, as regions closer to the tendon or bone may exhibit greater stiffness, and the local mechanical demands the tissue must withstand. If one area supports more stress than another, the fiber density must be higher.

### 3.3 Constitutive modeling

**FIGURE 7:**
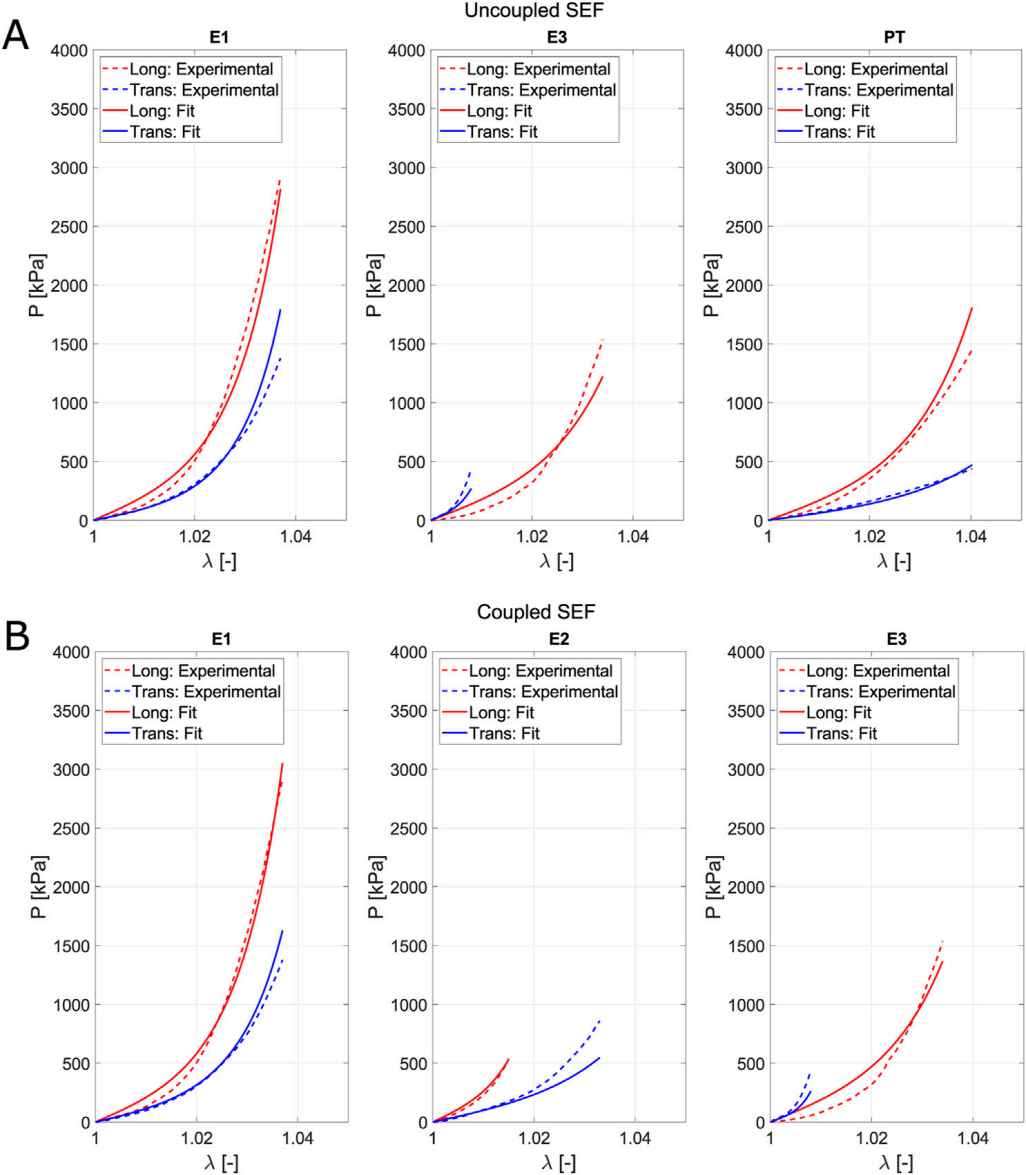
Fitting of the uncoupled model **(A)** and the coupled model **(B)** for the optimal combination of tests. Solid lines refer to the Cauchy stress from fitting, while the dash-dotted lines represent the mean Cauchy stress from experiments. Red lines indicate the longitudinal direction, and blue lines indicate the transversal.

Fitting is used to determine the parameters that define the model. It is based on a minimization problem where successive iterations of the parameters are performed until reaching a minimum in Equation 12. The objective of this step is to compare whether the uncoupled or coupled model is more appropriate based on their fitting and prediction capabilities. Figure 7 represents the average experimental curve for the fifth loading cycle (dashed lines) for each direction and the curves obtained from the fitting (solid lines) through the minimization process. Fitting accounts for the entire range of deformation reached in the different biaxial tests. However, for both the uniaxial and planar tension tests, the maximum values of λ only reach 1.04. Thus, all tests are fitted within the same range of deformation.

**FIGURE 8:**
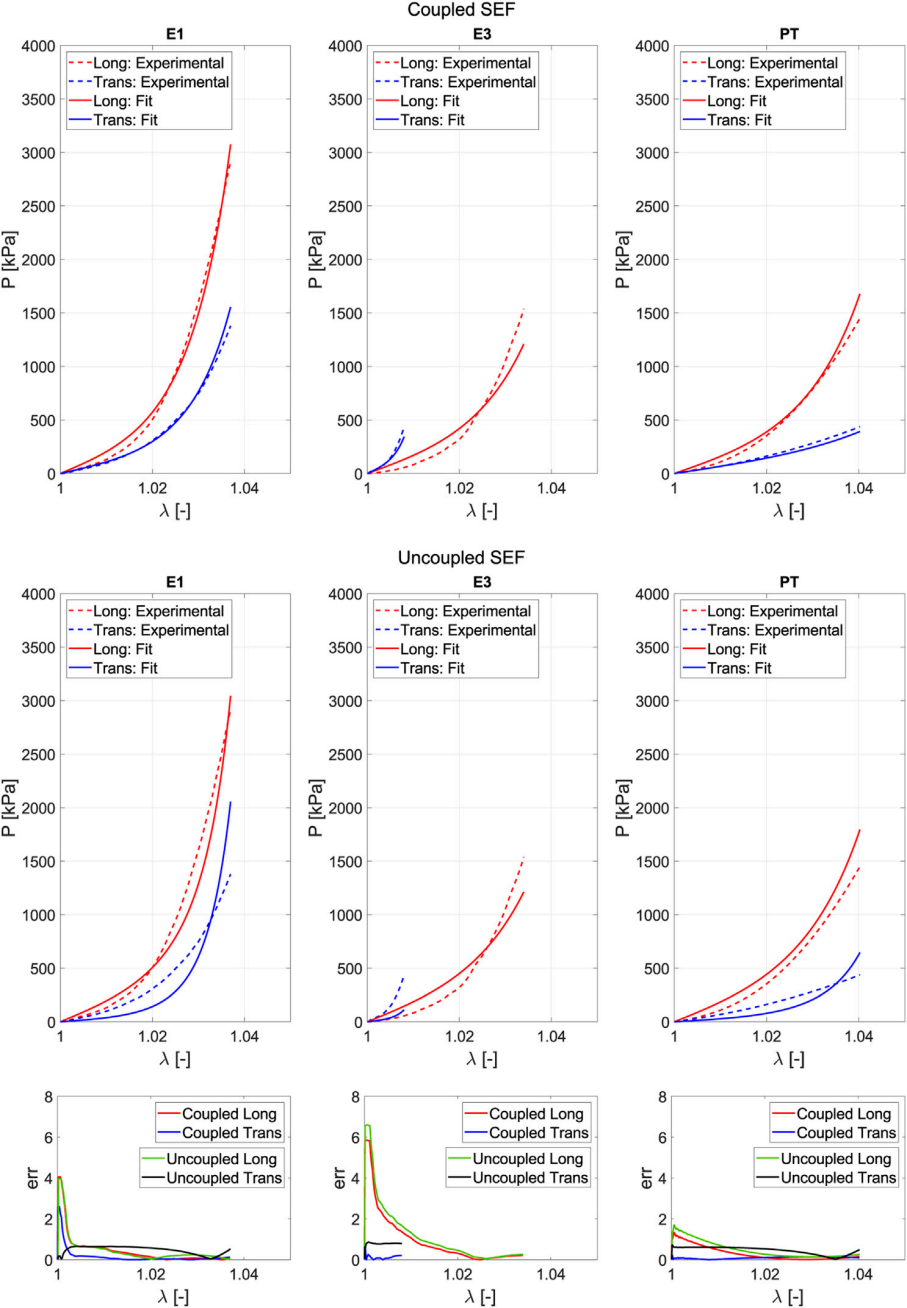
Comparison of the $err^{*}$ for the coupled and uncoupled models for the test combination with the best $R_{\mathrm{fit}}^{2}$ . The uncoupled model fitting was performed with constraints to ensure the parameters have physical meaning.

We derive the parameters for the fitting process by combining three tests. When the uncoupled model (based on Pancheri et al., 2014) was applied, the optimal combination of test with no constraints in the value of parameters was E1, E3 and PT (Figure 7A) with $R_{\mathrm{fit}}^{2}=0.964$ and $R_{\mathrm{pred}}^{2}=0.879$ . Regarding the structural parameters, the following values were obtained: $\mu_{\mathrm{iso}}=1470\;\text{kPa},\;\alpha =113.47,\;\mu_{l}=2442\text{kPa},\;k_{l}=167.19,\;\mu_{t}=0.00\;\text{kPa},\;k_{t}=0.01$ . We observe that the parameters associated with the family of transverse fibers are equal to zero, which is not physiologically plausible. If we consider the model as structural, there must be a relationship between the parameter and the tissue’s physiology. On the other hand, for the coupled model proposed in this work, the optimal combination was the ratios E1, E2, and E3 (Figure 7b) with $R_{\mathrm{fit}}^{2}=0.972$ and $R_{\mathrm{pred}}^{2}=0.878$ , the values of the structural parameters were $C_{0}=13.88\;\text{kPa},\;C_{1}=28.78,\;C_{2}=124.62,C_{3}=49.07$ . Unlike the uncoupled model, the parameter values in this case align with the expected structural function. The parameter associated with the longitudinal direction is greater than that of the transverse direction, and the latter is greater than that of the matrix. Comparing $err^{*}$ (see Equation 13) in both models for the maximum $R_{\mathrm{fit}}^{2}$ obtained with the uncoupled model (E1, E3, and PT), the coupled model exhibits lower relative errors, especially when the transversal direction is fitted, as shown in Figure 8. The longitudinal direction has a similar relative error in both models along λ , but it is slightly lower in the coupled model. The fitting for the uncoupled model was performed while considering constraints to ensure the physical meaning of the parameters. The relative error indicates that the proposed model achieves better results when different strain states are evaluated for soft-fibered tissues with fiber orientations close to 90°. The graphs show that the model better fits stress values for λ>1.005 . Note that $err^{*}=0$ means the model perfectly matches the experimental stress.

Observing the better fitting, improved prediction, and the physiological relevance of the parameters, the coupled model was chosen to study how the combination of tests affects the model’s predictive capability, considering its structural nature.

### 3.4 Constitutive model predictions

In this section, the combination of one to five tests is analyzed. It is essential to strike a balance between fitting and prediction. When parameters are obtained based on a single strain state, the fitting error is minimal, but the predictive capability is lost as the parameters become specific to that strain state.

Table 2 summarizes the material parameters and errors for each combination. Structural material parameters exhibit similar values, all within the same order of magnitude, except for the first fitting using only one test. As shown in Table 2, fitting becomes more challenging as the number of tests increases and the strain states become more diverse.

**TABLE 2:** Material structural parameters from fitting for the different combinations of tests using the proposed coupled strain energy function.

Combination	$C_{0}\;\left[\text{kPa}\right]$	$C_{1}\;\left[-\right]$	$C_{2}\;\left[-\right]$	$C_{3}\;\left[-\right]$	$R_{\mathrm{fit}}^{2}$	$\varepsilon_{\mathrm{fit}}$	$R_{\mathrm{pred}}^{2}$	$\varepsilon_{\mathrm{pred}}$
**E1**	**13.02**	**32.13**	**129.28**	**40.00**	**0.994**	**0.087**	**0.882**	**0,406**
E2	7.88	1.13. $10^{-5}$	230.96	155.52	0.999	0.032	0.719	0.686
E3	4.29	135.68	149.29	1.17. $10^{-5}$	0.995	0.089	0.620	1,084
UT	13.66	1.00. $10^{-5}$	147.36	36.72	0.990	0.128	0.499	0.951
PT	13.80	1.26. $10^{-5}$	129.99	61.49	0.995	0.071	0.442	0.843
E1, E2	16.42	1.00. $10^{-5}$	152.17	78.89	0.986	0.147	0.803	0.494
E1, E3	12.35	60.41	97.06	2.95	0.986	0.149	0.873	0.423
**E1, UT**	**12.57**	**40.87**	**124.76**	**24.09**	**0.989**	**0.126**	**0.854**	**0.471**
E1, PT	11.98	67.93	82.81	1.00. $10^{-5}$	0.992	0.106	0.846	0.452
E2, E3	4.89	67.96	203.04	120.29	0.988	0.125	0.576	0.971
E2, UT	9.03	86.77	124.90	11.69	0.945	0.270	0.740	0.688
E2, PT	9.36	90.82	78.25	12.47	0.958	0.199	0.856	0.472
E3, UT	7.74	73.24	148.30	11.56	0.978	0.191	0.830	0.590
E3, PT	9.41	94.38	81.30	1.00. $10^{-5}$	0.967	0.193	0.858	0.486
UT, PT	12.75	1.00. $10^{-5}$	144.51	52.57	0.968	0.206	0.364	0.994
**E1, E2, E3**	**13.88**	**28.78**	**124.62**	**49.07**	**0.972**	**0.211**	**0,878**	**0.371**
E1, E2, UT	14.08	37.39	116.67	29.28	0.973	0.202	0.870	0.412
E1, E2, PT	13.21	62.27	80.05	7.94	0.976	0.185	0.860	0.408
E1, E3, UT	11.59	62.27	129.16	24.98	0.983	0.166	0.844	0.492
E1, E3, PT	12.42	66.20	84.01	1.00. $10^{-5}$	0.983	0.157	0.846	0.454
E1, UT, PT	11.11	44.82	122.59	29.37	0.972	0.198	0.859	0.461
E2, E3, UT	7.00	90.18	142.75	26.59	0.953	0.258	0.710	0.739
E2, E3, PT	8.06	111.86	142.75	0.84	0.950	0.228	0.747	0.631
E2, UT, PT	13.25	30.94	116.83	39.75	0.917	0.317	0.894	0.369
E3, UT, PT	11.63	34.20	127.28	33.08	0.942	0.284	0.868	0.442
**E1, E2, E3, UT**	**13.02**	**39.60**	**120.63**	**30.52**	**0.969**	**0.222**	**0.861**	**0,417**
E1, E2, E3, PT	13.46	61.16	81.82	7.78	0.969	0.210	0.858	0.404
E1, E2, UT, PT	12.38	43.22	114.24	30.98	0.960	0.238	0.877	0.403
E1, E3, UT, PT	11.41	46.85	119.01	25.49	0.969	0.215	0.848	0.475
E2, E3, UT, PT	10.86	52.84	117.21	32.45	0.921	0.313	0.918	0.325
**E1, E2, E3, UT, PT**	**12.42**	**44.79**	**112.95**	**25.75**	**0.958**	**0.247**	**0.862**	**0,406**

Bold rows represent the best result of each combination.

Fitting with two strain states ( $R_{\mathrm{pred}}^{2}=0.854$ ; ε=0.471 ) implies losing precision when predicting tissue behavior for other strain states compared to fitting with three strain states ( $R_{\mathrm{pred}}^{2}=0.878$ ; ε=0.371 ). It should be noted that an excessive increase in the number of tests used for fitting does not necessarily result in an improvement in prediction error. While increasing from a single strain state to the combination of two may enhance prediction, fitting with four tests ( $R_{\mathrm{pred}}^{2}=0.861$ ; ε=0.417 ) does not yield a better prediction than fitting with three. In this sense, fitting with one strain state and with five strain states simultaneously was tested to corroborate the previous idea. Using only a single test, the E1 ratio yielded the best results in terms of the physiological meaning of the parameters and the prediction error $R_{\mathrm{pred}}^{2}$ that was equal to 0.882 with a ε = 0.406; however, the adjustment error $R_{\mathrm{fit}}^{2}$ was 0.994 with ε = 0.087. Using five tests, the fitting error $R_{\mathrm{fit}}^{2}$ is 0.958 with ε = 0.247, and the prediction error worsens with respect to the combination of three tests ( $R_{\mathrm{pred}}^{2}=0,878$ ; ε=0,371 ) with $R_{\mathrm{pred}}^{2}$ = 0.862 and ε = 0.406.

**FIGURE 9:**
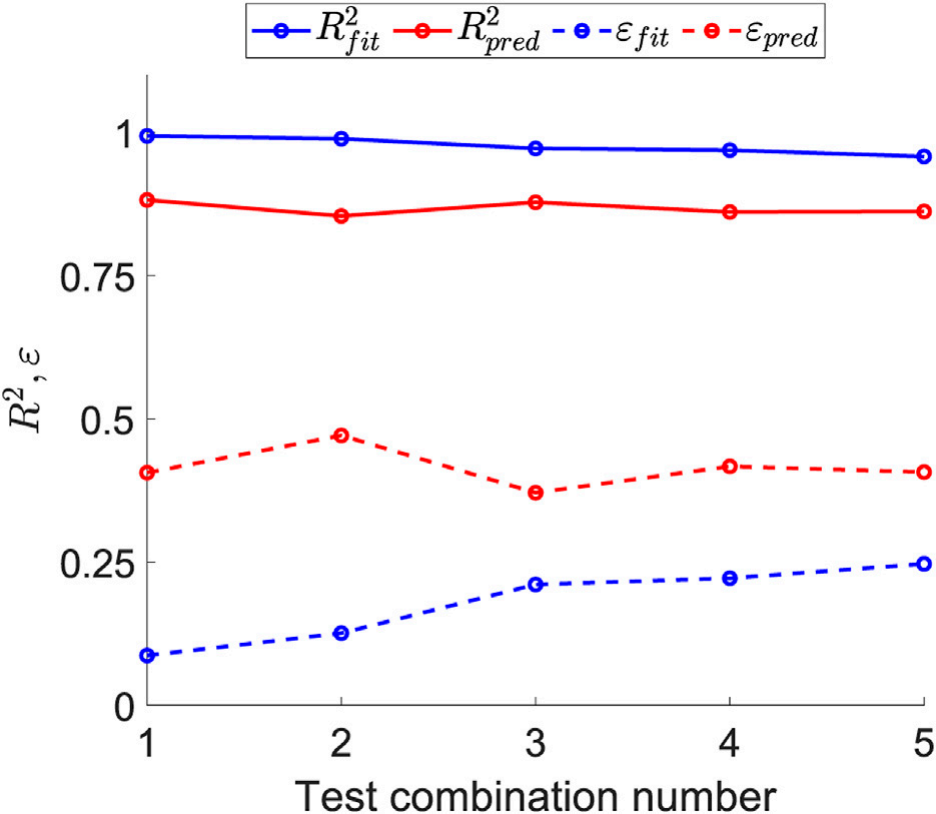
Sensitivity of the fitting and prediction errors as the number of tests used to obtain the structural parameters increases. $R^{2}$ and ε values correspond to the best result of each test combination.

Figure 9 illustrates the effect of the number of fitting tests on the errors in fitting and prediction. For each number of tests combined, the optimal prediction has been selected; that is, for the combination of three tests, the $R^{2}$ and ε values for the E1, E2, and E3 case are depicted.

**FIGURE 10:**
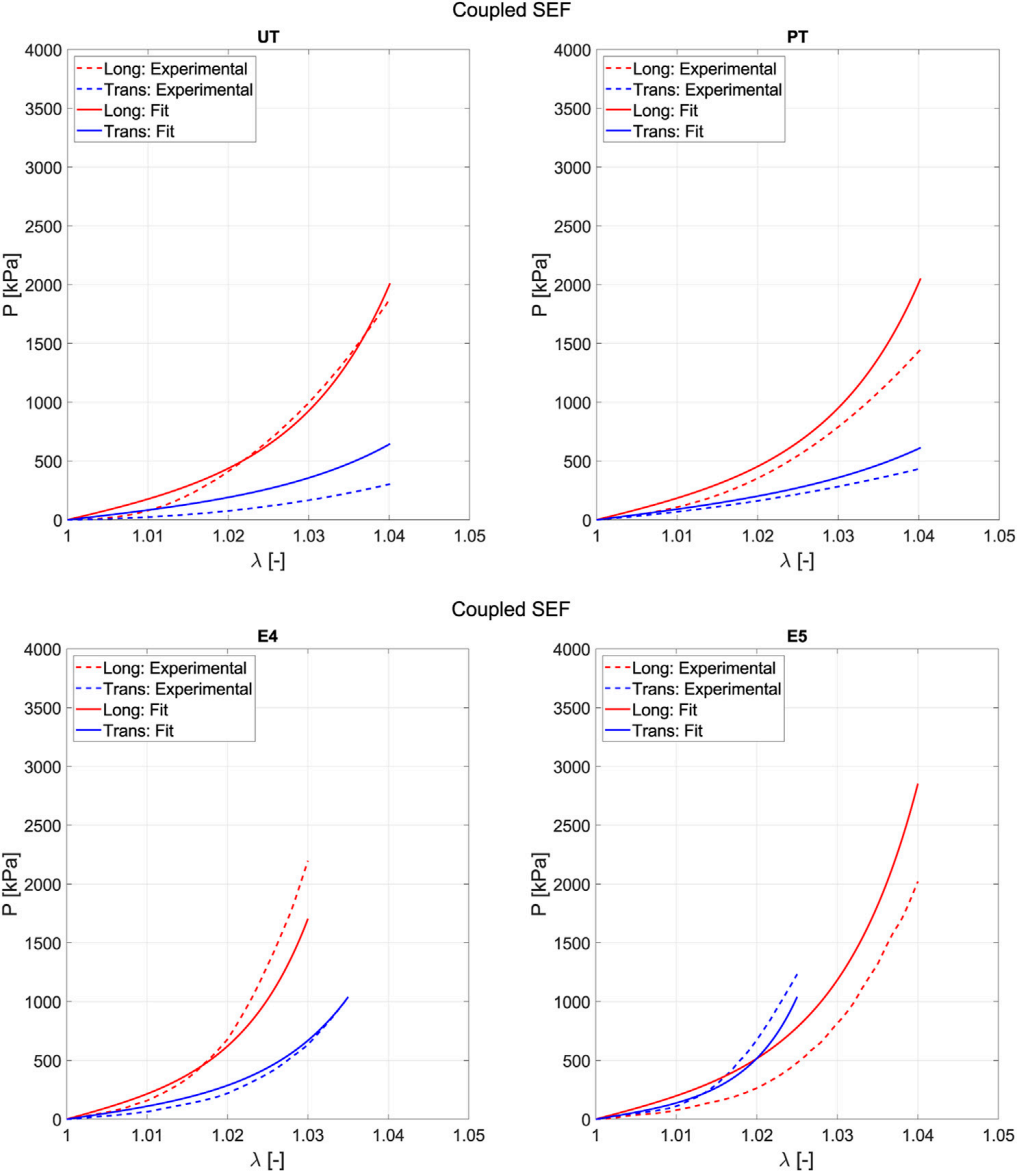
Prediction results when fitting is performed using three tests (E1, E2, and E3) with the proposed coupled SEF.

Figure 10 depicts the prediction curve for the tests that are not included when fitting with the three strain states (E1, E2, and E3). For low strain values, the prediction curve more accurately follows the real behavior experienced in the test. However, it is also observed that the biaxial ratio E5 proves challenging to predict because it represents a strain state that forces greater stiffness in the softer direction of anisotropy, which contradicts the tests used for fitting.

## 4 Discussion

Computational simulation is a powerful tool for studying and analyzing pathologies, treatments, and surgeries in the context of biomechanics. To achieve accurate results, an exhaustive characterization and the use of an adequate constitutive model capable of predicting tissue behavior are necessary. The fascia forms a continuous structure that can store approximately 20% of the total force produced by muscles (Blottner et al., 2019). Its stiffness is associated with plantar fasciopathy (Barreto Rabelo et al., 2023) and biomechanical responses (Cheung et al., 2004), among other functions. Computational simulation could help improve the understanding of its behavior and related pathologies. Despite its importance, the fascia remains an understudied tissue. For this reason, we have chosen fascia as the focus of our study.

### 4.1 Experimental remarks

Throughout this work, a multidimensional characterization has been presented, including three different tests that reproduce a wide range of strain states. The results show that fascia is a highly stiff tissue due to its structure, which consists of layers of collagen fibers spatially oriented in two directions. The highest deformation observed in our tests occurs in the plane tension test, reaching a maximum value of 7.5%. In the other tests, the maximum deformation reached is 5%. These elongation values are consistent with those reported in previous studies (Eng et al., 2014; Pancheri et al., 2014; Ruiz-Alejos et al., 2016). Fascia’s mechanical behavior is characterized by high stiffness, especially when compared to other soft tissues such as the myocardium and arteries. This stiffness allows the fascia to sustain high levels of stress with minimal strain, a characteristic typical of collagenous fibrous tissues like tendons. If tension increases by 8%–10%, it leads to visible tearing of tendon fibers, ultimately resulting in tendon rupture (Wang et al., 2012). The difference in stiffness between the longitudinal and transverse directions is related to the thickness and number of collagen fibers in each direction, which are greater in the longitudinal direction than in the transverse direction, as shown in the histological images in Figure 2A). Similar results were reported by Pancheri et al. (2014).

Eng et al. (2014) obtained 3.5 MPa in biaxial tests for a strain of 4%, while in our study, we measured 3 MPa for the same strain range. Pancheri et al. (2014) reported a maximum strain of 6% in biaxial tests and 8% in uniaxial tests. Regarding maximum stress values in uniaxial tests, they obtained 7 MPa for a strain level of 5.5%, whereas in our study, we reached 4 MPa at the same strain level. Comparing stress in biaxial tests, Pancheri et al. (2014) reported 3 MPa for a 4% strain, which matches our results. Additionally, Ruiz-Alejos et al. (2016) found that deep fascia exhibited a stress of 2.5 MPa at 5.5% elongation in uniaxial tests. Both results are within the same order of magnitude, with the difference accounted for by deviation. As observed by Pancheri et al. (2014), the data illustrate that specimens stretched along the longitudinally oriented fibers exhibit higher stiffness than those stretched in the transverse direction. Despite the different origins of the fascia samples, we observed similar values in sheep fascia lata to those reported by Stecco et al. (2013) for human crural fascia under the same stretch range. As seen in the literature and confirmed by our experimental results across different strain states, fascia exhibits high variability. The stress–strain curves presented in this work show that this deviation is consistent with that reported in other experimental studies.

### 4.2 Constitutive model remarks

**FIGURE 11:**
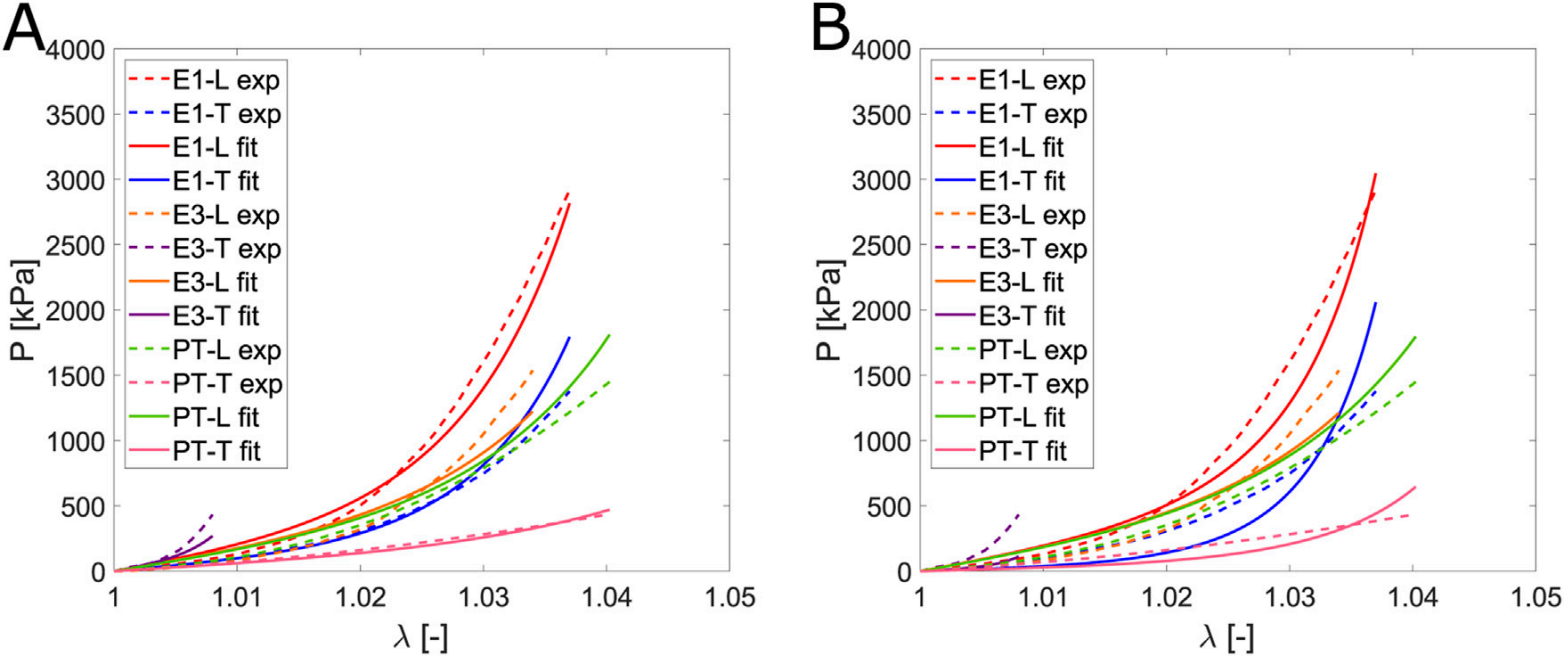
Comparison of the fit for the Pancheri et al. (2014) model: **(A)** without constraints and **(B)** with the constraint that the transverse parameters are greater than those associated with the matrix.

In this study, we evaluated the accuracy of the model proposed by Pancheri et al. (2014). As they described, a generic angle φ is used despite histological sections showing that collagen fibers form an angle between 80° and 90° (Stecco et al., 2009). We proposed a constitutive model based on a coupled strain energy function, assuming a 90° angle between the anisotropy directions representing the fiber orientations in the tissue. This assumption affects the choice of the constitutive model. Referring back to the formulation in Section 2.2, the unit vectors are defined as M={1,0,0} and N={0,1,0} , which, in turn, impacts the expressions used for stress calculation. In the model by Pancheri et al. (2014), the stress value in one direction does not depend on the other, as seen in the expressions for $\Psi_{1}$ , $\Psi_{4}$ , and $\Psi_{6}$ (Equation 8). Although the model can fit the experimental data (Figure 11A), issues arise with the obtained parameters, as they lack structural meaning. Specifically, the parameters related to the transverse fiber direction are reduced to 0, effectively neglecting one fiber direction. When we impose constraints in the minimization problem to ensure that the transverse parameters remain nonzero and greater than those associated with the matrix, the model is no longer able to fit the experimental data properly, as shown in Figure 11B.

To use an uncoupled constitutive model, it is necessary to not assume that the angle between the anisotropy directions is 90°. Instead, this angle becomes an additional parameter in the problem, defining the unit vectors as M={cos(φ),−sin(φ),0} and $N=\left\{cos\left(\varphi^{\prime }\right),sin\left(\varphi^{\prime }\right),0\right\}$ , where φ represents the fiber angle relative to the 1-axis, and thus $\varphi^{\prime }$ = 90°- φ . As stated in Pancheri et al. (2014), φ is a phenomenological parameter that they compare to the angle formed by fascia collagen fibers, despite describing a structural strain energy function (SEF). With the unit vectors defined in terms of sine and cosine, the analytical expression for stress calculation in one direction depends on the other. The model we propose in this work effectively fits the experimental data while assuming that the fibers form a 90° angle between them. This is because it incorporates both longitudinal and transverse contributions within the same exponential term, allowing stress in one direction to depend on the other. Even if the angle were treated as a parameter, our model could still accommodate it by incorporating it into the vector definitions, providing flexibility in considering different anisotropy angles.

Considering these aspects, the parameter fitting process was optimized using the coupled model proposed in this work. The main objective is to determine the minimal set of deformation states required for fitting in order to obtain accurate parameters that enable reliable predictions of fascia behavior with the fewest possible experiments.

Regarding the optimal combination of tests among the options studied and listed in Table 2, greater emphasis was placed on minimizing prediction error and reducing the number of test types required, as this directly impacts the number of samples and overall testing effort. As shown in Figure 9, which illustrates the variation of fitting and prediction errors with an increasing number of tests, both $R_{\mathrm{fit}}^{2}$ and $R_{\mathrm{pred}}^{2}$ stabilize and remain constant beyond three tests. This indicates that including more than three tests in the fitting process does not enhance prediction accuracy. Additionally, the three necessary tests—biaxial ratios E1, E2, and E3—belong to the same test type, reducing the number of specimens required and the overall testing time by eliminating the need for multiple testing machines.

The aim of a computational model is to enable simulations, making predictability a crucial factor. Our proposed coupled SEF demonstrates excellent predictability with only four parameters, considering that it accounts for three strain states. The material parameters we propose for characterizing fascia and predicting various strain states are listed in Table 3.

**TABLE 3:** Material parameters proposed for fascia characterization based on our SEF.

Combination	$C_{0}$	$C_{1}$	$C_{2}$	$C_{3}$
E1, E2, E3	13.88	28.78	124.62	49.07

Throughout this work, we have emphasized the importance of the obtained parameter values in relation to the structural nature of the model used for fitting. There must be coherence between these values and the structural components they represent. In this regard, it is possible to establish similarities with parameters from other studies. The parameters determined in this study represent a solution to a problem that does not have a unique solution. Therefore, direct comparisons of individual values to establish, for example, a stiffness criterion are not meaningful. Moreover, even if the two models are structural, their defining SEFs may differ. In fact, this work presents an SEF distinct from those proposed by Pancheri et al. (2014) and Ruiz-Alejos et al. (2016). Regardless of the absolute parameter values, a clear pattern emerges: parameters associated with the primary fiber direction are greater than those in the transverse direction. In turn, transverse parameters exceed those related to the isotropic component, which corresponds to the tissue matrix and lacks a mechanical function.

### 4.3 Limitations

This work has some limitations, one of which is that we tested samples from an animal model rather than human fascia. Although our results are similar to those obtained by Stecco et al. (2013), they cannot be directly extrapolated to the human model. Therefore, the parameters we propose should be used with caution in simulations for human studies.

Regarding the coupled SEF proposed in this study, as discussed by Anssari-Benam et al. (2024), the selection of classical invariants for the isotropic component may be suboptimal if $I_{2}$ is excluded, and similarly for the anisotropic component if $I_{5}$ and $I_{7}$ are not considered. The goal of this study is to develop a model that not only achieves a good fit but also enhances predictive accuracy across different deformation states while maintaining a straightforward formulation. To this end, we have chosen to use models that incorporate a simple exponential function and standard invariants commonly referenced in the literature.

Additionally, our model does not account for viscoelastic properties, which play a significant role in the behavior of soft tissues. The viscoelastic properties of fascia are typically analyzed through stress relaxation and dynamic mechanical analysis (DMA), both of which are widely documented in the literature (Bonifasi-Lista et al., 2005; Prevost et al., 2011; García et al., 2012; Calvo et al., 2014). These properties will be the subject of future studies. The perpendicularity of the fibers is considered; however, soft tissues exhibit fiber dispersion relative to the main direction. The next step to enhance the proposed model would be to incorporate a new parameter for dispersion using techniques such as polarized microscopy (Sáez et al., 2016). The mechanical behavior of soft tissues is governed by their underlying microstructure, particularly the extracellular matrix with embedded collagen fibers. Therefore, studying the micromechanical behavior of individual fibers can provide valuable insights into the macroscopic mechanical response. This approach is commonly used in microstructural models, where the behavior of individual fibers is represented and then homogenized by integrating over the surface of a sphere (Alastrué et al., 2009; Gasser, 2011; Weisbecker et al., 2015; Sáez et al., 2016). This work focuses on the macroscopic response and does not account for the micromechanical behavior of collagen fibers.

## 5 Conclusion

Characterizing soft biological tissues is challenging due to the many factors influencing accurate results. Tissue-related characteristics, such as heterogeneity, harvesting area, and inter-individual differences within the same species, as well as handling and testing protocols, can lead to variations across studies. Despite these considerations, our multidimensional characterization has yielded stress values that closely match those reported in the literature for the same strain levels.

This study highlights the importance of considering tissue characteristics and modeling assumptions when selecting an appropriate constitutive model. We assumed that fiber directions form an approximately 90° angle, which necessitates the use of a coupled constitutive model. An uncoupled model fails to properly fit the parameters under the condition that transverse parameters are neither 0 nor lower than the isotropic ones, as we consider the model to be structural. Furthermore, the uncoupled model lacks predictability, making it unsuitable for future simulations. These limitations motivated the development of the coupled SEF proposed in this work. Using this coupled model, we can accurately predict uniaxial, biaxial, and planar tension strain states with a single set of parameters.

Beyond proposing a new SEF that addresses the challenge of modeling anisotropic directions at 90°, we also analyzed the impact of the number of tests on fitting and prediction. Our results demonstrate that increasing the number of fitting tests does not improve the prediction of other strain states. Specifically, the biaxial ratios E1, E2, and E3 are sufficient to predict uniaxial, planar tension, and biaxial strain states.

The diversity of tests, the well-defined testing protocols, the experimental stress-strain curves, and their comparison with literature values, combined with the proposal of a new SEF and material parameters capable of predicting different strain states, provide a comprehensive and accurate understanding of the mechanical behavior of fascia. In addition to introducing a study on test combinations, this work offers valuable insights that contribute to a deeper understanding of fascia mechanics.

## 6 Statement of significance

Fascia is a collagen-rich soft tissue that has recently gained increasing importance in human physiology. Understanding its mechanical behavior is essential for comprehending its functions. To achieve this, we conduct a multidimensional characterization that includes different strain states. Additionally, we analyze two constitutive models: one widely used and another proposed in this study. Our findings highlight the importance of tissue structure when selecting an appropriate constitutive model. The primary goal of a constitutive model is to accurately predict strain states, which depends on the material parameters obtained through fitting. Therefore, this study also explores the combination of mechanical tests to optimize the fitting process.

## Data Availability

The raw data supporting the conclusions of this article will be made available by the authors, without undue reservation.
